# Stable isotope analysis suggests nutrient connectivity between salmon and kelp within a commercial scale open coast integrated multi-trophic aquaculture system

**DOI:** 10.1038/s41598-026-45539-5

**Published:** 2026-03-26

**Authors:** Amalia Krupandan, Lynne Falconer, Julie Maguire, Deirdre McElligott, Rona A. R. McGill, Trevor Telfer

**Affiliations:** 1 Institute of Aquaculture, University of Stirling, Stirling, FK9 4LA Scotland, UK; 2 Bantry Marine Research Station Ltd., Gearhies, Bantry, Co. Cork Ireland; 3 National Environmental Isotope Facility, Scottish Universities Environmental Research Centre, Scottish Enterprise Technology Park, East Kilbride, G75 0QF UK

**Keywords:** IMTA, Marine, Nitrogen, Nutrient transfer, Seaweed, Sustainability, Ecology, Ecology, Environmental sciences, Ocean sciences

## Abstract

This study explores nutrient connectivity between Atlantic salmon (*Salmo salar*) and sugar kelp (*Saccharina latissima*) within a commercial-scale integrated multi-trophic aquaculture (IMTA) system in Bantry Bay, Ireland. In this system the kelp farm is located next to a salmon farm that restarted production in mid-2023 following four years of no production. This allowed a comparison between baseline conditions at the same site during a kelp-only production season in 2023, prior to salmon farming, and a salmon-kelp production season in 2024. Fieldwork campaigns were conducted during each year to collect samples of kelp, water, and proxy nutrient sources (fish feed, faeces, particulates, seaweeds). Stable isotope analysis and a Bayesian mixing model were used to assess uptake of nutrient sources by the kelp. The *δ*^15^N values differed between the study period of each year, with consistently lower *δ*^15^N values observed during 2024. Mixing model estimates showed a pronounced shift after salmon farming commenced. Comparative growth data between years revealed increases in kelp blade length, width, wet weight, and tissue nitrogen content during 2024. The findings suggest nutrient transfer from salmon farming to the kelp, with stable isotope analysis serving as a complementary method to growth and water sampling for determining nutrient connectivity.

**Supplementary Information:**

The online version contains supplementary material available at 10.1038/s41598-026-45539-5.

## Introduction

Integrated Multi-Trophic Aquaculture (IMTA) is increasingly promoted as a sustainable approach to finfish aquaculture, promoting bioremediation of farm effluents through the co-culture of low-trophic species near finfish farms^1–4^. Fish-seaweed IMTA, particularly with kelp, offers potential benefits by mitigating the environmental impact of finfish farming and enhancing production of seaweed grown for commercial harvest as food, industrial raw materials, and biofuels^5^. However, purposeful commercial-scale implementation remains limited partly due to uncertainties about nutrient connectivity between fish and seaweed. The environmental and economic success of IMTA depends on the degree of this connectivity, with no connectivity offering few benefits, limited connectivity supporting growth, and high connectivity providing both growth and nutrient remediation. These different outcomes have distinct implications for the management and regulation of IMTA^6^.

Sugar kelp (*Saccharina latissima*) is a well-studied species^7^ and a prominent candidate for IMTA, particularly with Atlantic salmon (*Salmo salar*), due to its high nitrogen demand and tolerance of high wave action^8,9^. Kelp primarily acquires nitrogen in dissolved forms, with nitrogen being the most limiting nutrient for growth^10^. In natural ecosystems, kelp obtains nitrogen through vertical mixing, atmospheric nitrogen fixation, terrestrial runoff, and microbial regeneration^11^. Other macroalgae may also constitute a nutrient source for kelp as tissue erodes through wave action and releases organic exudates^12^. However, in an IMTA system, kelp could benefit from dissolved inorganic nitrogen (DIN) from salmon metabolic waste, primarily excreted as ammonia across the gills^13^, and to a lesser extent as urea, which decomposes into cyanate and ammonium^14^. In addition, uneaten feed^15^ and faeces^16,17^ decompose into particulate organic matter (POM), which is remineralized by bacteria into dissolved forms like ammonium^18,19^. Ammonium can then be oxidized to nitrite and nitrate by nitrifying bacteria^20^. Kelp preferentially absorbs ammonium due to its lower energy cost for assimilation^11^, though it can assimilate both ammonium and nitrate simultaneously, switching between sources based on external concentrations^21^. Nitrate is utilized during rapid growth phases when nitrogen demand exceeds supply^10^.

Within IMTA systems, unknowns in nutrient connectivity also persist due to difficulties with establishing trophic connectivity between species in dynamic open-water environments^22,23^. Directly assessing nutrient connectivity in the field is particularly challenging for inorganic extractive species like kelp, where traditional diet studies are not applicable. Other studies have assessed nutrient connectivity relating to kelp conceptually, through hypothetical model scenarios^23,24^, through models based on field and experimental data^25–27^; ex situ studies^28–31^, and in situ field studies^32,33^. Studies frequently infer nutrient uptake through growth measurement, measuring photosynthetic rate, and biochemical changes in the seaweed that may suggest uptake of excess nutrients^29,32,34^, and/or extensive water sampling to determine differences in nutrient levels between IMTA and non-IMTA production^33,35^. These methods are useful but limited, as it is difficult to establish a direct link between the farm derived nutrients and their uptake by kelp due to both the transient nature of dissolved wastes from cage aquaculture^36^ and the high phenotypic plasticity and adaptability of kelps to different environmental conditions^9,37,38^. Accordingly, the changes in water chemistry or kelp physiology may not be due to the presence of excess nutrients derived from fish farming.

Biochemical markers can be used to more directly trace the impacts of fish farm wastes^39^. Stable isotope analysis has been applied to determine trophic connectivity between species by comparing isotopic signatures of prey tissue from different sources to that of a consumer species^40^. In aquaculture it has been more recently applied to trace the nutrient flow of fish farm wastes from open water fish farms to extractive species in IMTA systems^41^. While several IMTA isotope studies have investigated the nutrient connectivity of fish farms to benthic deposit feeders^42,43^ and suspension feeders^44,45^, fewer studies have looked at nutrient connectivity and uptake within open-water fish-kelp IMTA systems^46–48^. In hydrographically complex environments, stable isotope analysis allows the uptake and assimilation of salmon wastes to be directly investigated, rather than inferred by ex situ studies, indirect methods or models.

This study focused on an incidental commercial-scale IMTA system in Bantry Bay, Ireland, where a salmon farm and a kelp farm are located approximately 200 m from each other. In the 2023 kelp production cycle, and the previous 4 years, only the kelp farm was in operation, with the salmon farm restarting full scale production before the 2024 kelp production cycle. This allowed a comparison between baseline conditions at the same site during a kelp-only production season in 2023 and a salmon-kelp production season in 2024. This difference allowed us to compare kelp growth and nutrient uptake at the same site before and after the introduction of salmon farming, reducing the effect of high spatial variability in environmental conditions at a highly dynamic site and providing a baseline to assess the salmon farm’s impact. The aim of the study was to establish if nutrient connectivity between the two farms occurred and to determine if the kelp was utilising C and N derived from the salmon effluent and investigate whether this contributed to an increased growth of seaweed.

## Results

### Environmental measurements

**Fig. 1:**
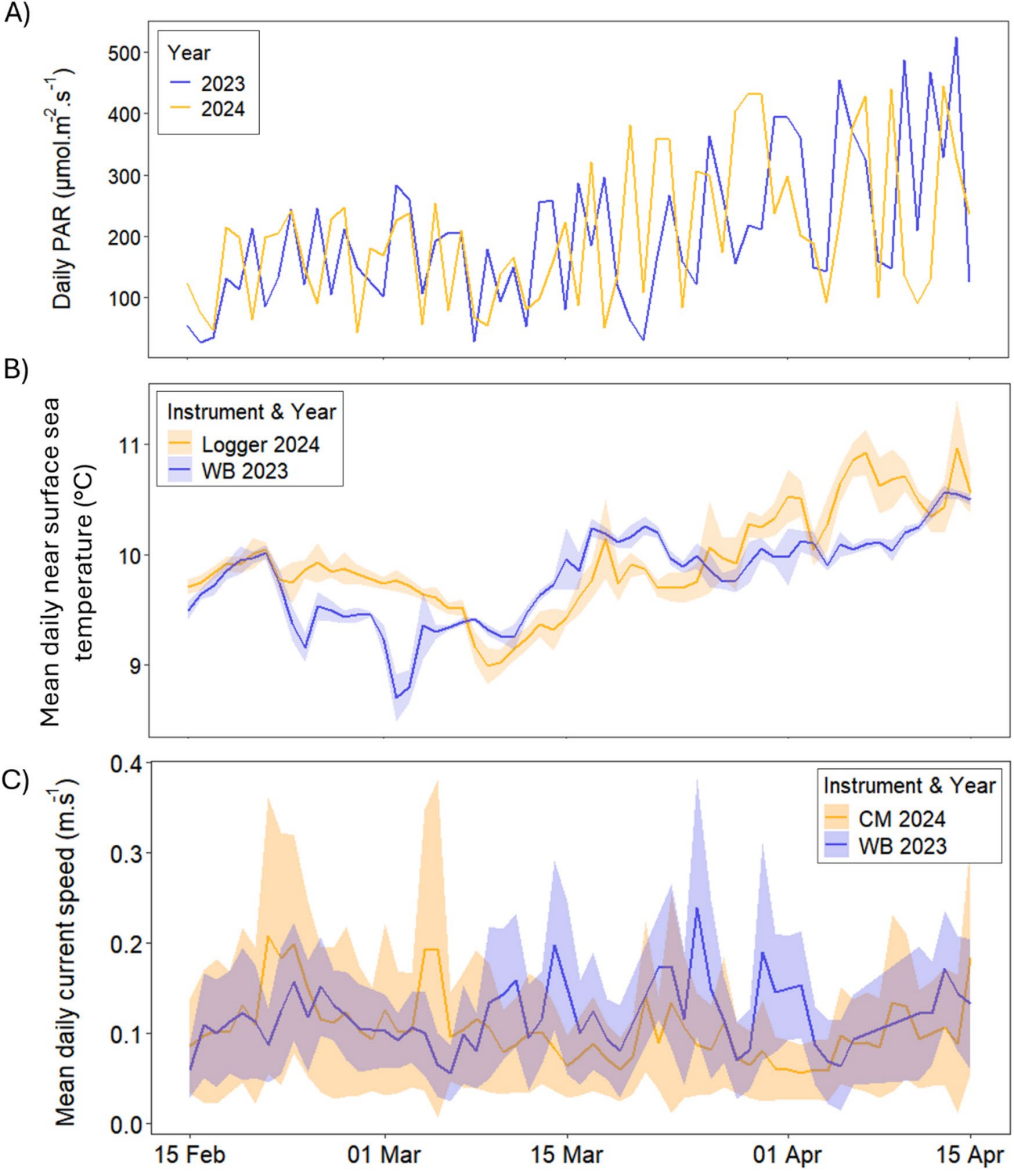
Environmental conditions in Bantry Bay from mid-February to mid-April for 2023 and 2024. (**A**) Satellite-derived daily photosynthetically active radiation (PAR) measurements from NOAA-20 VIIRS. (**B**) Mean daily near-surface sea temperature (°C) with 2023 data sourced from the Bantry Bay wave buoy (WB 2023) and 2024 data sourced from a temperature logger (Logger 2024) deployed at the kelp farm. (**C**) Mean daily current speed, with 2023 data from the Bantry Bay wave buoy (WB 2023) of the Irish Marine Data Buoy Observation Network (Marine Institute) and 2024 data from the current meter deployed at 7 m depth at the kelp farm (CM 2024). Standard deviation indicated by envelope.

Mean values for environmental measurements across the study period can be found in Table 1. Daily PAR levels were similar between the study period in each year (Fig. 1A). Although derived from different instruments, current speeds were comparable between years (Fig. 1B). Temperature trends were similar across both periods, with fluctuations throughout and slight increases from March to April (Fig. 1C). While 2023 showed broader temperature variation, this may reflect differences in data sources. For both periods, current direction was consistently recorded at N/NW, moving from the salmon farm towards to northern portion of the kelp farm.

Measured DIN concentrations did not differ between sampling points on the kelp farm (Table 1). Measured mean nitrate concentrations were found to be significantly higher in the 2024 kelp production period than the 2023 period, there were no significant differences found in other mean surface water nitrogen concentrations between the years due to large variability in the results (Table 1). Overall, measurements taken in 2023 showed more variability compared to 2024. Nitrate concentrations were elevated in February 2024 and were observed to be lower later in the production cycle (Table S1, Supplementary Material).

**Table 1:** Mean ± SD of measured DIN concentrations within the kelp farm during the kelp production cycle of each year. Results of one-way ANOVAs showing differences in DIN concentrations between sampling points over both years. Results of Independent Sample T-tests showing differences in DIN concentrations between years. For each sampling period *n* = 30.

Nutrient	Mean concentration (± SD)		Difference between sampling points	Difference between years
	2023 sampling period	2024 sampling period		
NH_4_^+^ (µg L^− 1^)	187.32 ± 270.93	87.50 ± 67.87	F(1,4) = 0.986, *P* = 0.4238	t(1,58) = 1.958, *P* = 0.055
NO_2_^−^ (µg L^− 1^)	2.98 ± 1.52	2.33 ± 1.26	F(1,4) = 0.102, *P* = 0.98.13	t(1,58) = 1.824, *P* = 0.073
NO_3_^−^ (µg L^− 1^)	75.86 ± 55.12	120.15 ± 71.53	F(1,4) = 0.345, *P* = 0.8463	t(1,58) = -2.686, *P* = 0.009
TN (µg.L^− 1^)	266.16 ± 285.12	209.97 ± 109.27	F(1,4) = 0.955, *P* = 0.440	t(1,58) = 1.008, *P* = 0.318

### Stable isotope analysis of kelp nutrient sources

**Fig. 2:**
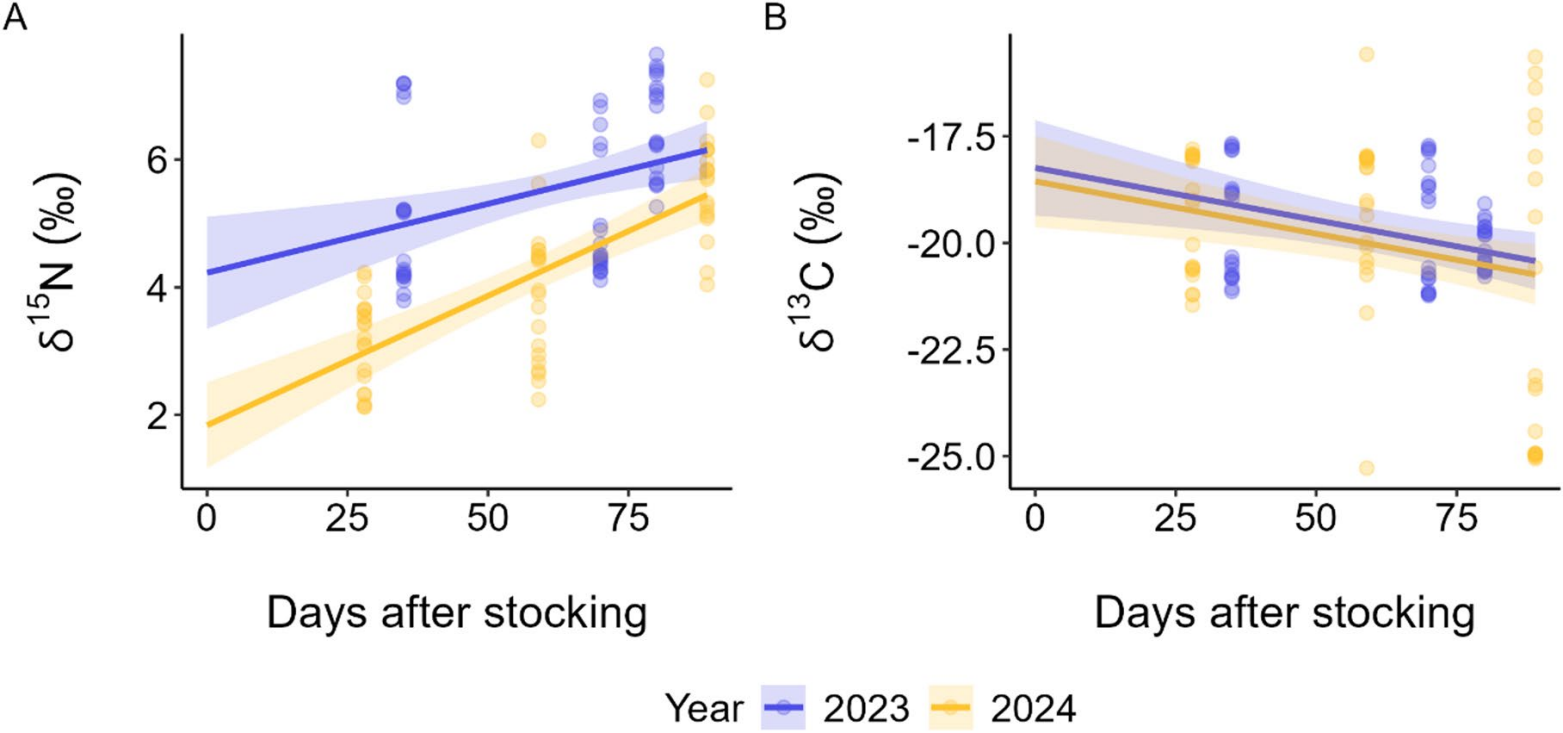
Observed values (points) and fitted ANCOVA trajectories for (**A**) δ^15^N and (**B**) δ^13^C by year against days after stocking (DAS). No salmon farm was present during the 2023 kelp production period (blue); present in the 2024 production period (yellow). Shaded envelopes are 95% confidence bands for the mean. For δ^15^N the model was year × DAS and slopes differed between years. For δ^13^C the model was year + DAS with a common linear trend and an adjusted year difference.

**Fig. 3:**
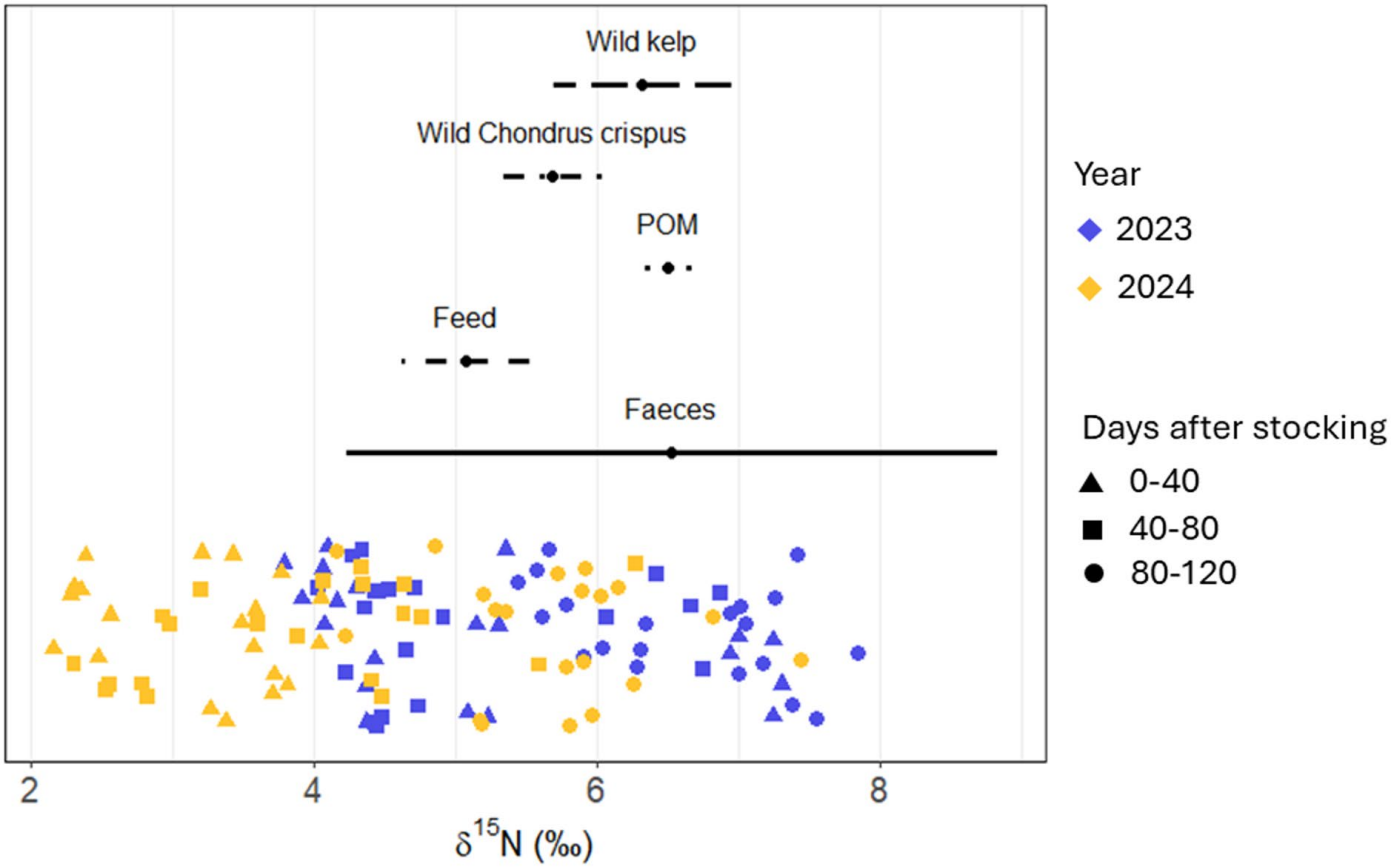
*δ*^15^N ranges of farmed kelp during the 2023 (blue, no salmon farm) and 2024 (yellow, salmon farm) kelp production periods, and dissolved nitrogen sources (wild kelp, wild *C. crispus*, POM, fish feed and fish faeces). Shapes indicate time from kelp farm stocking that samples were taken; 0–40 days (triangle); 40–80 days (square); 80–120 days (circle).

The stable isotope analysis of *δ*^15^N showed significant differences between production periods of each year (Fig. 2A). An ANCOVA revealed that fitted linear functions for *δ*^15^N differed between years, when accounting for days after stocking (DAS) of the kelp as a covariate (Type II ANOVA, year × DAS: F(1,116) = 4.68, *p* = 0.033). ANCOVA-derived slopes indicated enrichment over time in both years, but with different magnitudes. In 2023, δ^15^N increased by 0.022‰ d^−1^ (95% CI 0.008–0.035), while in 2024 it increased by 0.041‰ d^−1^ (95% CI 0.030–0.051). The range of *δ*^15^N values in 2023 (4‰ to 8‰) overlapped with natural nitrogen sources such as wild kelp, *C. crispus*, and POM (Fig. 3). The wide range of *δ*^15^N in farmed kelp during the 2023 period suggests exposure to other nitrogen sources, potentially including sources with a similar isotopic signature to fish wastes, despite no active fish farms nearby. For reference, *δ*^15^N values in fish feed were 4.5‰ to 5.5‰, while fish faecal waste ranged from 4‰ to 9‰. During the 2024 period, the *δ*^15^N values in farmed kelp were more dispersed, with some samples as low as 2‰. Figure 3 also shows that *δ*^15^N values of farmed kelp in 2023 show no grouping based of DAS, whereas in 2024 *δ*^15^N values are lower earlier on in the production period and revert to 2023 levels after 80 DAS. In contrast, an ANCOVA revealed no evidence for a year difference after adjusting for DAS for δ^13^C (Type II ANOVA, year: F(1,117) = 0.75, *p* = 0.390) (Fig. 2B). δ^13^C decreased with days after stocking (Type II ANOVA, DAS: F(1,117) = 8.89, *p* = 0.0035). No further investigation into the contributions of different carbon sources to kelp growth was undertaken due to the consistency in *δ*^13^C values.

### MixSIAR Bayesian mixing model: estimated N source proportions (δ^15^N)

**Fig. 4:**
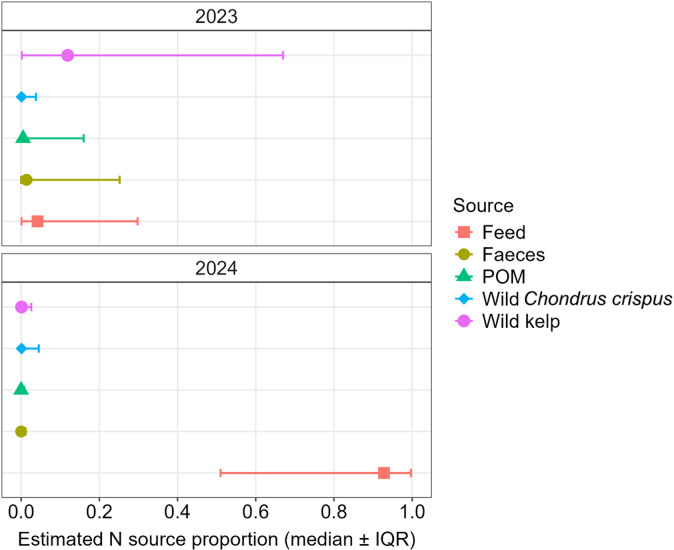
MixSIAR-estimated median N source proportions for farmed kelp during the 2023 and 2024 kelp production cycles. Error bars show the interquartile range (IQR; 25th−75th percentiles). Considered DIN proxy sources were fish farm wastes (feed and faeces), POM, wild *Chondrus crispus*, and wild kelp (*Saccharina latissima*, *Alaria esculenta*, *Laminaria digitata*, *Laminaria hyperborea*).

For the 2023 kelp production period MixSIAR estimated the median and interquartile range (IQR; 25th–75th percentiles) of source proportions required to reproduce the observed farmed kelp δ^15^N values as: wild kelp 11.9% (IQR 0.2–67.0%), fish feed 4.2% (IQR 0.1–29.8%), fish faeces 1.4% (IQR 0.0–25.2%), POM 0.5% (IQR 0.0–16.0%), and wild *Chondrus crispus* 0.1% (IQR 0.0–3.8%) (Fig. 4). For the 2024 kelp production period, estimated proportions were: fish feed 92.8% (IQR 51.0–99.7%), fish faeces 0.0% (IQR 0.0–1.1%), POM 0.0% (IQR 0.0–0.8%), wild kelp 0.1% (IQR 0.0–2.6%), and wild *C. crispus* 0.1% (IQR 0.0–4.5%) (Fig. 4). Interquartile ranges for several sources overlapped, particularly in 2023, indicating substantial uncertainty in the estimated proportions.

### Kelp growth measurements and C:N composition

**Fig. 5:**
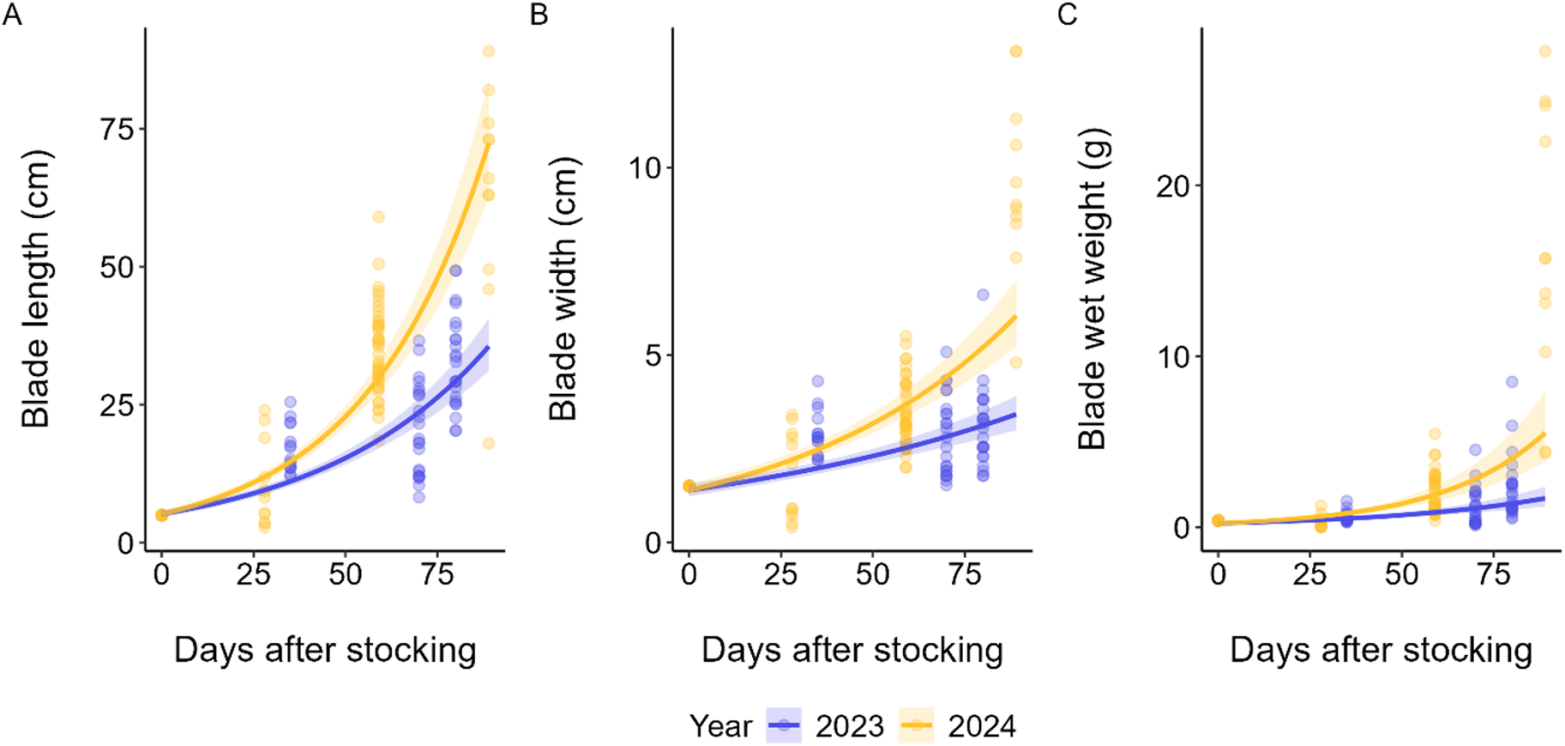
Observed values (points) and fitted ANCOVA trajectories for average (**A**) blade length (cm), (**B**) blade width (cm), and (**C**) blade wet weight (g) of farmed kelp by year against days after stocking. Shaded envelopes represent 95% CI.

Kelp biomass increased substantially over the production period of both years, but 2024 exhibited markedly faster growth (Fig. 5). ANCOVAs on log-transformed variables revealed significant year × DAS interactions for length (Type II ANOVA: F(1,154) = 54.5, *p* < 0.001), width (F(1,154) = 34.33, *p* < 0.001), and wet weight (F(1,154) = 23.76, *p* < 0.001), indicating that fitted growth trajectories differed between years. ANCOVA-derived slopes expressed as daily percentage growth confirmed these differences (Table 2): change in all growth variables over time were greater in the 2024 period compared to 2023.

**Table 2:** Estimated mean daily growth rate derived from fitted ANCOVA models of log-transformed kelp growth data collected in February–April of 2023 and 2024.

	Mean daily growth (% d^−1^) derived from fitted ANCOVA model	
	2023	2024
Blade length	2.18 (95% CI 1.95–2.41)	2.99 (95% CI 2.75–3.25)
Blade width	1.02 (95% CI 0.79–1.24)	1.66 (95% CI 1.42–1.91)
Blade wet weight	2.23 (95% CI 1.66–2.80)	3.59 (95% CI 2.96–4.22)

**Fig. 6:**
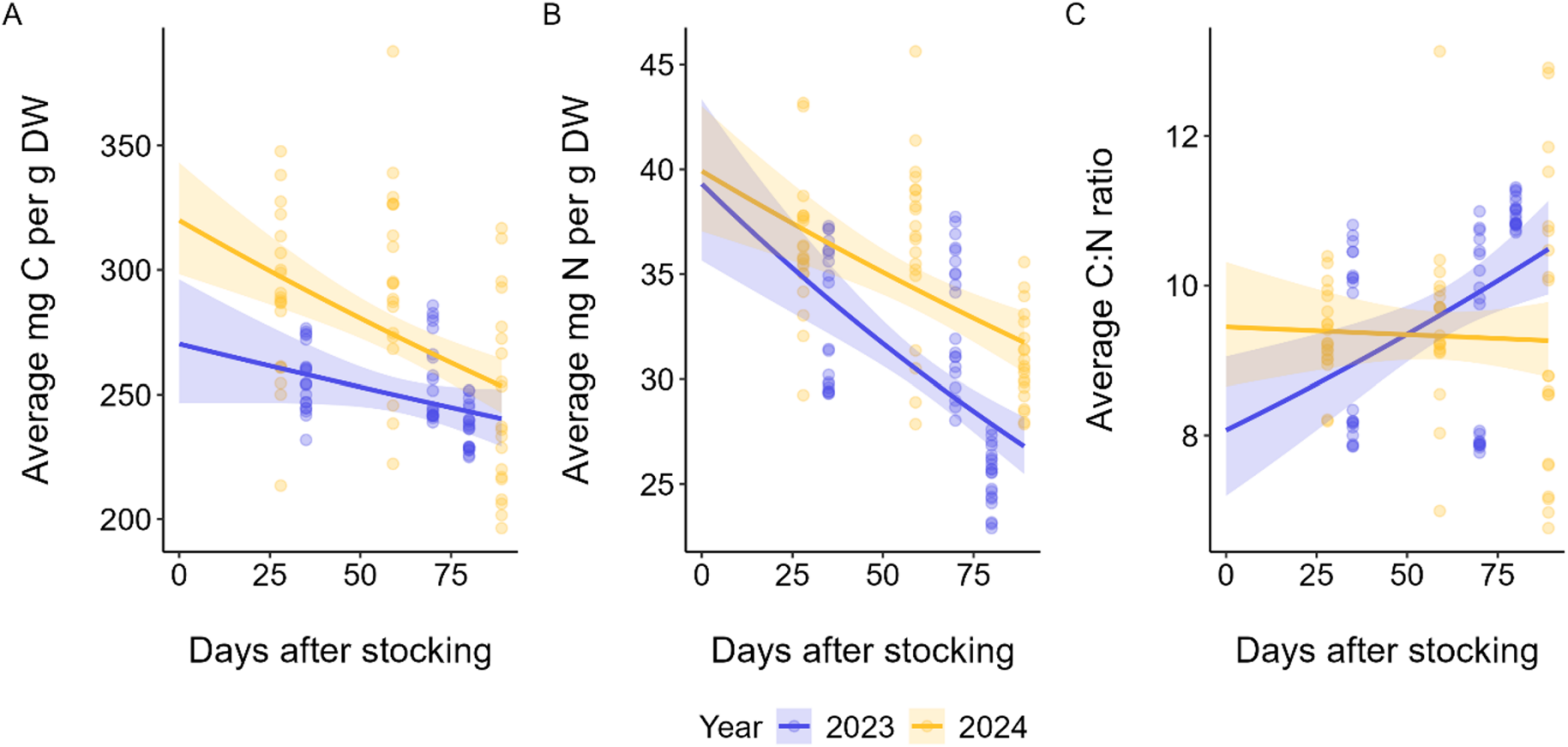
Observed values (points) and fitted ANCOVA trajectories for average (**A**) carbon content (mg C. g DW^− 1^) and (**B**) nitrogen content (mg N. g DW^− 1^) and (**C**) molar C:N ratio of farmed kelp by year against days after stocking. Shaded envelopes represent 95% CI.

Carbon and nitrogen content declined over the production period in both years, with 2024 maintaining higher absolute values throughout. An ANCOVA on C content revealed no significant year × DAS interaction (F(1,116) = 2.03, *p* = 0.16), but significant main effects of both year (F(1,116) = 20.62, *p* < 0.001) and DAS (F(1,116) = 23.23, *p* < 0.001). An ANCOVA N content showed strong main effects of year (F(1,116) = 32.39, *p* < 0.001) and DAS (F(1,116) = 46.62, *p* < 0.001), with a marginally non-significant year × DAS interaction (F(1,116) = 3.13, *p* = 0.079). Therefore, both C and N content were significantly higher in 2024 but did not significantly differ in the temporal trends across years (Fig. 6A,B).

Throughout the 2024 production cycle, the C:N ratio remained relatively stable over time, whereas in 2023 the C: N ratio increased from 9.14 to 10.96 between 70 and 80 days since stocking (Fig. 6C). An ANCOVA on log-transformed C:N revealed a significant year × DAS interaction (F(1,116) = 7.59, *p* = 0.007), indicating divergent temporal trajectories between years.

## Discussion

This study adds to the growing body of evidence that kelp placed in the vicinity of fish farming show increased growth and nutrient assimilation^32,46,48^. We also demonstrate that while traditional IMTA assessments (e.g., growth, water quality, and biochemical composition) provide insights into kelp’s nutrient uptake, stable isotope analysis can help evaluate whether kelp nitrogen signatures shift in a manner consistent with aquaculture influence, thereby supporting interpretation of growth and biochemical responses in proximity to fed aquaculture.

Monitoring nutrient concentrations from salmon farm waste streams is challenging due to both short- and long-term variability^36^, which complicates the assessment of nitrogen dynamics. Ammonium fluxes in the water column are highly dynamic and unreliable for assessing nitrogen dynamics unless observing high nutrient loads or monitoring at fine temporal scales^49^. Nitrate, being more stable in seawater, may serve as a better indicator of DIN inputs at broader scales^50^. Elevated nitrate levels were observed in February 2024, when the salmon farm was active, but declined towards the end of the kelp production period. This pattern may reflect a combination of two processes acting together: reduced input during fish starvation and harvest in April 2024, and a surge in kelp growth in March 2024 that likely depleted ambient nitrogen below 2023 levels. This also points to the huge difficulty in assessing nutrient uptake in situ through water sampling alone, as high inherent variability does not reveal trends. Paired with growth and tissue data, the findings are slightly clearer. Nitrate is the principal externally supplied nitrogen form supporting storage in *S. latissima*^11^, and prolonged availability enables reserve building^51^. Here, higher tissue N and a relatively stable C:N ratio while the fish farm was active indicate sustained N supply during the 2024 cultivation period. Elevated nitrate February 2024 plausibly allowed kelp to accumulate reserves that supported later growth under favourable temperature and PAR, whereas lower nitrate in February 2023 limited storage and subsequent growth potential; kelp then likely drew on internal reserves when external N became scarce^11,52,53^. This interpretation aligns with observations of earlier, larger growth peaks of *S. latissima* near salmon farms^48^ and helps explain the similar final lengths (~ 70 cm) reached in 2024 when kelp was cultured adjacent to farming.

Stable isotope analysis elucidates this further. During the 2023 kelp cultivation period, *δ*^15^N values of kelp overlapped with isotopic signatures of natural nitrogen sources, such as wild macroalgae and POM, as well as nitrogen sources resembling fish farm wastes. As no fish farm was present in the 2023 kelp cultivation period, overlap with farm wastes could be attributed to high and low δ^15^N sources unaccounted for in this study. Elevated *δ*^15^N values may suggest contributions from anthropogenic sources like sewage or runoff from agricultural livestock, known for high *δ*^15^N levels^54^. However, the ranges of *δ*^15^N for several marine N sources are reported as^55^: less than 0‰ for atmospheric N sources, −3 to 3‰ for industrial fertiliser, 4 to 8‰ for ambient marine N sources, and above 10‰ for treated wastewater. This both confirms that the *δ*^15^N values observed in this study for both sources and farmed kelp are within ambient ranges, except for a low-*δ*^15^N influence shown in farmed kelp earlier in the 2024 kelp production season. Although the overlap in *δ*^15^N among sources and their consistency with ambient values makes it difficult to determine provenance, this low δ^15^N suggests a shift in nitrogen origin for farmed kelp, potentially influenced by the fish farm. Earlier observations^46^ have recorded *δ*^15^N values for *S. latissima* near salmon farms ranging from −4.8‰ to 3.3‰, which is more similar to the low-*δ*^15^N values observed earlier in the 2024 kelp production season. Fish feed had the lowest *δ*^15^N value compared to other sources, and the influence of the fish farm is supported by the observation that *δ*^15^N reverts to 2023 levels in April of 2024—after the salmon farm had begun its harvest process and fish starvation. When observing the MixSIAR model results, fish feed-derived N stands out as a plausible nutrient source for farmed kelp in the 2024 cultivation period. Because several sources overlap in δ^15^N, MixSIAR cannot uniquely resolve all source proportions and small non-zero estimates (particularly in 2023) should be interpreted cautiously. For instance, the large variation in recorded fish faeces δ^15^N means that any influence from this source cannot be determined. Nevertheless, the between-year contrast was marked for fish feed contributions to farmed kelp δ^15^N: median estimated were substantially higher during the 2024 period compared to 2023, with no overlap in interquartile range. This pattern is consistent with a stronger feed-associated N signal in the 2024 cultivation period, while precise apportionment among the remaining sources remains uncertain.

While previous studies have typically observed ^15^N enrichment near fish farms^56,57^, several factors in this study may explain the observed lower *δ*^15^N values. Organic fish feeds often include marine protein by-products, such as fish processing trimmings^58^, which likely exhibit *δ*^15^N variability based on species, geographic origin, and processing methods. The feed in this study contained ~ 40% marine protein from trimmings and likely also reflects modern feed formulations that incorporate more terrestrial plant oils and proteins than in the past^59–61^. Historical comparisons^46^ also show that ^15^N in fish feed has declined over time, further corroborating the lower *δ*^15^N in this study. Isotopic discrimination during fish excretion also likely contributed to the lower *δ*^15^N. Fish excrete nitrogen as ammonium or urea, which is isotopically lighter than their tissue or feed due to selective retention of the heavier isotope in anabolic processes^62–64^. Hydrolysis of urea to ammonium results in minor isotopic fractionation^65^. Organic N leached from uneaten feed pellets similarly undergoes remineralisation into ammonium. This ammonium can undergo nitrification, where lighter nitrogen isotopes are preferentially oxidised, resulting in nitrate with reduced *δ*^15^N^54,66–69^. Early in the 2024 kelp growth season, fish-farm-derived ammonium could have underwent nitrification and contributed to the elevated nitrate available for kelp uptake, characterised by a distinct low-*δ*^15^N signature. Isotopic discrimination during nitrogen uptake and assimilation by kelp could further explain the observed low-*δ*^15^N values. Studies on other macroalgae (e.g., *Acanthophora spicifera* and *Enteromorpha intestinalis*) report negligible isotopic discrimination during uptake under low nitrogen conditions^70,71^. However, studies have noted that other photoautotrophs have shown that under higher N availability, ^14^N was preferentially assimilated and a lower *δ*^15^N is observed in the plant tissue^70,72^. This may be the case for *S. latissima* in high-nutrient aquaculture environments where the preferential uptake of the lighter isotope during periods of higher DIN enrichment was observed^46^. Additionally, it has been reported that in plants, pathways of assimilation for both nitrate and ammonium preferentially utilise ^14^N, but only in periods when nutrient demand is lower than supply^68^. Nutrient analyses of this study’s site show that background DIN levels (in absence of the fish farm) are high, and thus this area could not be considered low nutrient. This is supported by the observed C:N ratios of the farmed kelp. C:N ratios of *S. latissima* have been shown to be lower in high-nutrient conditions^9,73^. The ratios observed in this study are both lower than those previously reported by Grebe et al. (9.4 to 23.4)^55^ and Zhu et al. (13 to 40)^9^ suggesting little nitrogen limitation. The addition of extra nitrate from the fish farm suggests that kelp here can selectively take up lower *δ*^15^N DIN. Taken together, isotopic fractionation during transformation and uptake processes mean that any fish-farm signal in kelp δ^15^N is expected to reflect processed DIN rather than the δ^15^N of bulk feed or faeces, and this should be considered when interpreting mixing-model estimates.

The need to use proxy DIN sources highlights a key limitation in this study, and a knowledge gap concerning using stable isotope analysis for macroalgae in the marine environment. In mixing models, the differences in isotopic signature of consumer species and their prey is typically accounted for by trophic discrimination factors (TDFs), reflecting the level of discrimination against specific isotopes (e.g., ^15^N or ^13^C) between organisms at different trophic levels^74^. While TDFs have been established for consumer species tracking isotopic transfer up trophic levels, they have not been calculated for marine autotrophs, as fractionation is generally assumed to be minimal^75^. Nitrogen from fish feed is altered before assimilation by kelp through fish excretion, bacterial processing (oxidation), and uptake by kelp. Kelp therefore do not directly uptake nitrogen from feed (e.g., DON leached from feed) but from nitrogen processed through various metabolic and biogeochemical cycles, representing a nitrogen source not directly measured in this study but derived from fish farming. While the isotope fractionation during these processes may explain the lowered *δ*^15^N in 2024 compared to feed, this effect has not been explicitly measured. The MixSIAR outputs thus should not be taken as exact quantitative apportionment of kelp nitrogen among organic waste materials, but can highlight where further corroboration is needed for potential nutrient connectivity pathways. We highlight the need for accurate fractionation information for both dissolved nutrient cycling and kelp uptake processes in future isotope studies of kelp IMTA systems. As shown by Grebe et al.^55^, the influence of environmental variables and physiological processes on kelp *δ*^15^N in situ is challenging to unravel.

This study demonstrates that salmon farming coincided with an increase in nitrate levels and kelp growth within an adjacent kelp farm. Stable isotope analysis indicated minimal change in kelp δ^13^C patterns between cultivation periods, whereas δ^15^N signatures shifted in 2024, with mixing-model estimates placing greater weight on the fish feed category, highlighting a potential farm-associated N-enrichment pathway. The study also underscores the need for further research into isotopic fractionation during the remineralisation of farm wastes in dissolved form and their subsequent uptake by kelp. Improved understanding of these processes would increase the precision of using isotopes as tracers for bioremediation in dynamic environments, supporting robust governance of open-water IMTA.

## Methods

### Study site

**Fig. 7:**
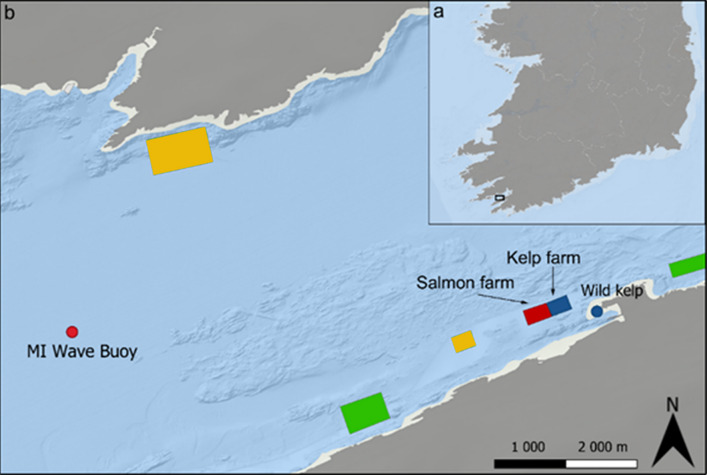
(**a**) map of Ireland, showing study location in Bantry Bay; (**b**) study site in Bantry Bay, consisting of salmon farm (red block) and adjacent kelp farm (blue block). Nearby aquaculture concessions shown as green (Blue Mussel) and yellow (Atlantic Salmon) blocks; wild kelp beds indicated by blue circle; Marine Institute data buoy indicated by red circle. Map created using QGIS Version 3.40.5^76^. Data sources: Esri Ocean Basemap^77^; NUTS 3 regional boundaries^78^; Aquaculture Sites dataset from the Marine Institute (Ireland)^79^.

The study site was located in Bantry Bay, a coastal inlet in the southwest of Ireland, characterised as an exposed location subject to storms. The site included a kelp farm and an adjacent salmon farm (Fig. 7). The depth of the bay at the production site was approximately 20 m. The kelp farm covers 60,000 m^2^ licensed area where 11 lines, each 110 m long, were deployed at surface level (approximately, 0–1.5 m depth). In both 2023 and 2024, ropes seeded with ~ 5 cm long S. *latissima* sporelings were added to these lines on 16th January, with harvesting taking place in May-June, depending on the growth rate of the kelp. Harvesting is scheduled before the summer to prevent the degradation of kelp caused by fouling epiphytes.

The salmon farm covers 64,000 m^2^ licensed area and consists of three net-pen enclosures of 40 m diameter and 18 m depth down to the cone. Each pen yields 250 tons of salmon and requires 250 tons of feed. The salmon farm is an organic farm site, which, in Ireland, requires a smaller stocking density, feed derived from certified ingredients (organic, sustainable), and no use of antifoulants. This farm is smaller than many commercial salmon farms (e.g. a site in Norway could produce > 4000 tonnes), as it functions as a conditioning site where adult fish are grown to market size, with an average stocking weight of 1.21 kg and an average harvest weight of 5.57 kg live weight. The salmon farm operated from July 2023 to April 2024 and was fallow for four years prior. Throughout the salmon production cycle, fish were fed once per day in the morning—except from April 2024 when the salmon farm began its harvesting process, during which fish were starved. The harvesting process occurred over the first two weeks of April 2024. The kelp and salmon farms were directly adjacent to one another, with the kelp farm approximately 200 m downstream of the salmon farm. The nearest additional salmon farms were located approximately 900 m to the southwest (also an organic farm, not active during 2023) and 5 km to the northwest of the study site. Access to the kelp farm and the adjacent salmon farm, and permission to conduct sampling at these facilities, was granted by the respective farm operators prior to fieldwork.

### Environmental measurements and water sample collection

**Fig. 8:**
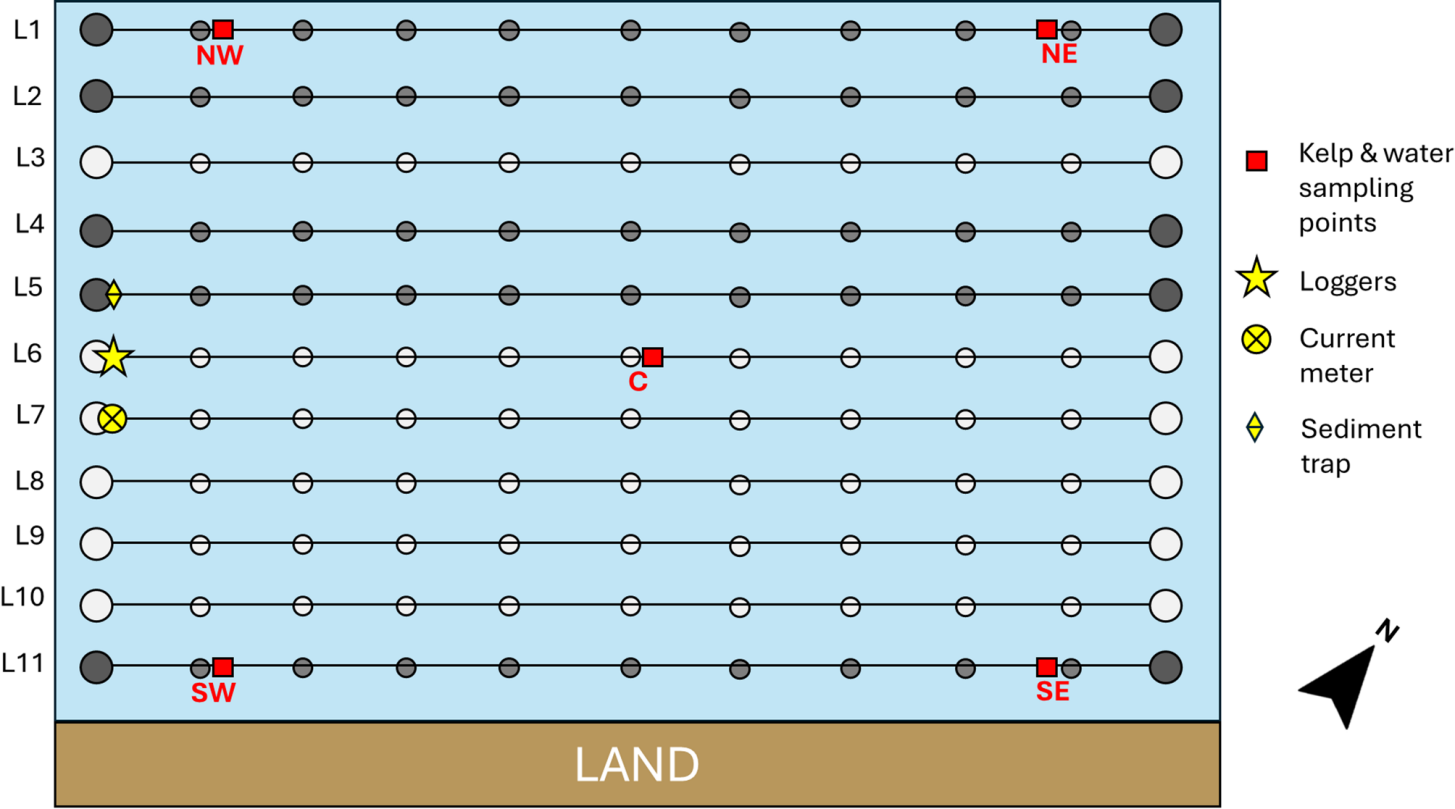
Detailed sampling layout of kelp farm. Grey circles indicate buoys making up kelp lines (large: mooring buoy; dark grey: intact line; light grey: line damaged by storms in 2024). Red blocks indicate kelp sampling points. Yellow star indicates location of PAR and temperature loggers. Yellow circle indicates location of current meter. Yellow diamond indicates location of sediment trap. Salmon farm located 200 m to the west.

Temperature and current speed data were obtained from the Irish Marine Data Buoy Observation Network^80^ from a data buoy (Fig. 7). For light energy (as Photosynthetically Active Radiation, PAR), satellite-derived PAR measurements for Bantry Bay from the NOAA-20 VIIRS (Visible Infrared Imaging Radiometer Suite)^81^ were used for production periods of both years (15 February to 15 April). This PAR data had a 4 km spatial resolution. In 2024, several additional loggers were deployed due to the termination of the data buoy measurements in Bantry Bay. These included a pH and temperature logger deployed at 1 m depth on the mooring buoy of line 6 (Fig. 8), and an electromagnetic current meter at 7 m depth on the south-westerly mooring buoy of the line 7 of the kelp farm (Fig. 2). An additional PAR meter was deployed at 1 m depth on line 6 as well, to validate satellite data. The current meter measured current speed, direction, and water temperature through burst sampling every 20 min, with each burst lasting 5 s. The pH/temperature logger recorded data every 5 min, while the PAR meter conducted 1-min burst sampling every hour.

Water sampling was conducted at specific intervals expressed as days since stocking. In 2023, water samples were collected on Day 35 (20th February), Day 70 (27th March), and Day 80 (6th April). In 2024, water samples were taken on Day 28 (13th February), Day 59 (15th March), and Day 89 (14th April). Sampling dates were planned for even intervals to capture growth stages consistently; however, this was not always possible due to adverse weather conditions. Sampling occurred at five points (NE, NW, SE, SW and C) on the kelp farm (Fig. 8). At each sampling point, two water samples were collected at 20 cm depth in 500 ml HDPE Nalgene bottles that had been autoclaved and rinsed with 10% HCl solution to remove carbonates. Each sample bottle was pre-rinsed in seawater at the location before sampling. Water samples were filtered to remove particulates (particle retention > 11 μm) and stored at − 20 °C until analysis. Samples were defrosted at room temperature overnight before analysis using standard methods for TAN (analytical standard: EPA-148-C), NO^−^ _2_ and NO^−^ _3_ (analytical standard: EPA-126-C). At each sampling period and year, a one-way ANOVA was performed to determine if nutrient concentrations differed between sampling points. These tests showed no difference in nutrient concentration between sampling points (Table 1), thus were pooled so that only sampling month and year were considered as factors.

### Kelp collection and growth measurements

Samples of farmed kelp were collected in February, March and April in 2023, and in the same months of 2024 after the adjacent salmon farm had been stocked in July 2023. Sampling occurred at the same dates and times as water sample collection. Farmed kelp sampling occurred at the same five points as water samples (Fig. 8). At each point, five plants were randomly selected and cut from the seaweed line at the stipe, using a knife or scalpel. Storms damaged 6 of the 11 kelp lines in March of 2024, removing sampling point C for that year (Fig. 8). Due to a necessity for increased statistical power, more kelp samples were taken from other points in 2024, thus samples were pooled so that sampling point was not included as a factor for analysis. The collected plants were placed in polyethylene sample bags and immediately processed. Before weighing and measuring, the plants were lightly scrubbed with a nylon-bristle nail brush to remove any epiphytes and rinsed with milli-q water. The length of each plant was measured with a tape measure, from the start of the blade to the end, with eroded blades measured up to the eroded edge. The width was recorded at the widest section of the blade. After air drying the plants for five minutes between double layers of absorbent tissue paper, each was weighed individually using an analytical balance accurate to four decimal places. Following the measurements, the plants were divided into meristem and distal tissue sections and stored at − 20 °C until freeze-drying. Freeze-drying was chosen due to its minimal effect on the isotopic signatures of carbon and nitrogen^82^.

The effect of both year and sampling period on growth could not be statistically assessed, as sampling periods occurred at slightly different times along the kelp growth cycle each year. To assess the effect of year on kelp growth while accounting for variation due to time, an analysis of covariance (ANCOVA) was conducted. Prior to ANCOVA application, growth variables (blade length, width, and wet weight) were log-transformed to linearize the data, and Levene’s tests were performed on each year’s data to ensure homoscedasticity. Days after stocking (DAS) was included as a covariate to control for its influence on growth, and year and the interaction between year and DAS were treated as fixed factors. Type II sum of squares was used to assess significance, and model assumptions were verified using residual diagnostics. Distal and meristem tissues were pooled for these analyses to improve precision of year and DAS effects, because tissue type did not alter the δ^15^N-DAS or δ^15^N-year relationship (ANCOVA and type II ANOVA; tissue × DAS: F(1,122) = 0.02, *p* = 0.881; year × tissue: F(1,122) = 0.63, *p* = 0.427). All analyses were performed in R version 4.4.1^83^ using the package ‘car’^84^.

### Nutrient source sample collection

Source categories (fish feed and faeces, trap-collected POM, and wild macroalgae) were treated as proxies for fish farm waste-derived and background nitrogen signatures, recognising that kelp assimilates nitrogen primarily as dissolved inorganic nitrogen after transformation of organic wastes.

Settling POM was collected through a surface-tethered sediment trap with four collection cylinders^43^ placed on the south-westerly mooring buoy of line 5 of the kelp farm, adjacent to the salmon farm (Fig. 8). The sediment trap was deployed in mid-March 2024 and retrieved one month later to accumulate sufficient material for stable isotope analysis at this dynamic, low-accumulation site. Trap-derived isotope results are interpreted only for the trap deployment/overlap period. After retrieval of the sediment trap, the integrated collection bottles were removed and stored at 3 °C until processing on the same day. Collection bottles were rinsed with milli-q water and contents were then filtered through a 25 μm nylon mesh filter. The residue was collected and weighed after processing by whole-sample centrifugation^85^. Samples were stored at − 20 °C until freeze-drying.

In March 2024, five 120 g samples of commercial salmon feed were obtained from the salmon farm. The feed was used on the farm for at least 2 months prior to sampling, thus was representative of the feed ingested by the salmon. Salmon faeces were collected from 5 fish from the salmon farm in March 2024 (all fish at the salmon farm were of the same cohort, and stocked at 1.2 kg average body weight in June of 2023). The fish were sacrificed by fish farm operators during routine monitoring operations of the fish farm, and faeces were extracted. Feed and faecal samples were stored at − 20 °C until freeze drying.

In mid-March 2024, samples of wild macroalgae were collected from intertidal shores approximately 3.8 km southeast of the salmon farm (Fig. 7). Species collected were the most abundant macroalgal species found in the region: *S. latissima*, *Alaria esculenta*, *Laminaria hyperborea*, *Laminaria digitata*, and *Chondrus crispus*. Approximately 250 g of each species was collected, with 10 plants collected per species. Within each species, plants of the same size and condition (not degraded) were collected. During collection, kelp species were intentionally cut above the meristem to ensure that plants could re-grow over time. Samples were kept chilled until processing on the same day. Plants were rinsed with milli-q water, lightly scrubbed using a nylon-bristle nail brush to remove any epiphytes and stored at -20 °C until freeze drying. These macroalgal sources were divided into “wild kelp” and “wild *Chondrus crispus*”.

### Stable isotope analysis

Farmed kelp, POM, fish faeces, fish feed and wild macroalgae samples were sent for bulk *δ*^13^C and *δ*^15^N stable isotope analysis, also yielding carbon and nitrogen content measurements, and the C/N ratio. Sample preparation involved freeze-drying, grinding, and encapsulation. Freeze-dried samples were first homogenized using a spice grinder, followed by further homogenization in a pestle and mortar. Lipids were not removed from feed samples as lipid extraction itself can also affect the *δ*^13^C & *δ*^15^N^86–88^. Lipid extraction was not deemed necessary for POM, algal tissue and faeces samples because of their low overall lipid content. The ground samples were then packaged into tin capsules (5 × 3.5 mm) based on the mass required for carbon and nitrogen detection, which was determined through test analyses of the different sample material. Between samples, all apparatus, including spatulas and forceps, as well as working surfaces, were cleaned with 90% ethanol to avoid contamination. The packaged samples were stored in 96-well plates at 3 °C until they were ready for analysis at the National Environmental Isotope Facility at Scottish Universities Environmental Research Centre, East Kilbride, Scotland. Samples were combusted in an elemental analyser (Elementar vario PYRO cube) to produce CO_2_ and N^15^ gases, which were then directed to a mass spectrometer (Thermo Finnigan Deltaplus XP IRMS) for isotope measurement. Lab standards (Gel, Alagel, Glygel) and the international reference material USGS40 (L-glutamic acid) ensured measurement accuracy. The isotopic composition (*δ*X) was calculated using the equation:

$$\:\delta\:X=\left(\frac{{R}_{Sam}}{{R}_{Ref}}-1\right)\times 1000 \%$$

where R_Sam_ is the isotopic ratio (e.g., ^13^C/^12^ or ^15^ N/^14^N) of the sample, expressed relative to the isotope ratio of international reference materials VPDB for carbon and N_2_ (air) for nitrogen.

Similarly to kelp growth, ANCOVAs were performed to assess the effect of year (2023 vs. 2024) on kelp nutrient content (carbon content, nitrogen content, C:N ratio) and stable isotope values (*δ*^13^C, *δ*^15^N). DAS was included as a covariate to account for temporal variation. For all variables, Type II sum of squares was used to assess significance, and model assumptions were verified using residual diagnostics. All analyses were performed using R version 4.4.1^83^ and the ‘car’ package^84^.

To explore whether farmed kelp δ^15^N values were more consistent with nitrogen derived from salmon-farm waste pathways, we used the MixSIAR Bayesian mixing model^89^. This model was applied separately for nitrogen and carbon as kelp absorb these nutrients through independent pathways. MixSIAR is a mass-balance mixing model and uses Markov chain Monte Carlo to sample the range of plausible source mixtures whose weighted isotope values could reproduce the observed farmed kelp isotope values, given the specified sources and model settings. The mixture dataset contained isotopic ratios for farmed *Saccharina latissima* with year as a factor, which was treated as a random effect. Source data were loaded as raw isotopic ratio observations. No TDFs were applied as fractionation during waste transformation and kelp uptake is not well defined for this system; applying an arbitrary TDF could bias estimates. Outputs are therefore interpreted conservatively as conditional on the specified source categories and the assumption of no net fractionation. Three independent sampling runs (“chains”) were used; each chain produced 1,000,000 iterations. The model used an uninformative prior on source proportions. The median value and the interquartile range (Q1 to Q3) of each source’s contribution was reported from the probability distributions. Model convergence was assessed with Gelman-Rubin diagnostics.

## Supplementary Information

Below is the link to the electronic supplementary material.

Supplementary Material 1

## Data Availability

Krupandan, A. (2025). Data relating to publication: Stable isotope analysis suggests nutrient connectivity between salmon and kelp within a commercial scale open coast integrated multi-trophic aquaculture system (Version 1) [Data set]. Zenodo (10.5281/zenodo.14718615).
