# Supplementary material for: Stable isotope analysis suggests nutrient connectivity between salmon and kelp within a commercial scale open coast integrated multi-trophic aquaculture system
Source: Sci Rep. 2026 Mar 26;16:15135. doi: 10.1038/s41598-026-45539-5 (PMC13172352; doi:10.1038/s41598-026-45539-5)
Supplement: Supplementary file 1 — Supplementary Material 1 [file 41598_2026_45539_MOESM1_ESM.pdf]

Supplementary Material for Stable isotope analysis suggests nutrient connectivity between salmon and kelp within a commercial scale open coast integrated multi-trophic aquaculture system

Amalia Krupandan[1*], Lynne Falconer[1], Julie Maguire[2], Deirdre McElligott[2], Rona A.R. McGill[3], Trevor Telfer[1]

1. Institute of Aquaculture, University of Stirling, Stirling, Scotland, FK9 4LA, UK.

2. Bantry Marine Research Station Ltd., Gearhies, Bantry, Co. Cork, Ireland.

3. National Environmental Isotope Facility, Scottish Universities Environmental Research Centre, Scottish Enterprise Technology Park, East Kilbride G75 0QF, UK

Correspondence: amalia.krupandan@stir.ac.uk

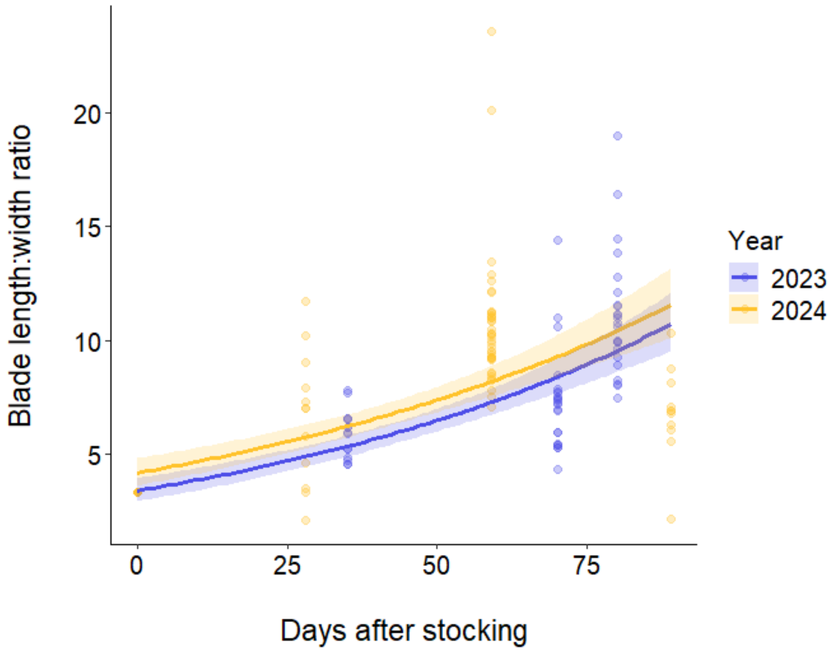

Figure S1: Observed values (points) and fitted ANCOVA trajectories for length to width ratio of farmed kelp by year against days after stocking. Shaded envelopes represent 95% CI.

Table S1: Mean ± SD of measured DIN concentrations within the kelp farm during the kelp production cycle of each year, at each sampling time point (measured as Days After Stocking, DAS).

**Nutrient**	**2023**			**2024**		
	35 DAS	70 DAS	80 DAS	28 DAS	59 DAS	89 DAS
NH_4^+ (µg.L^{-1})	290.13 ± 432.38	137.87 ± 111.45	133.96 ± 139.74	83.86 ± 61.85	114.49 ± 87.16	64.15 ± 44.58
NO_2^- (µg.L^{-1})	2.32 ± 0.40	3.76 ± 0.97	2.86 ± 2.28	2.37 ± 0.28	3.76 ± 0.50	0.85 ± 0.22
NO_3^- (µg.L^{-1})	54.75 ± 37.97	97.40 ± 31.75	75.42 ± 79.56	168.73 ± 42.89	163.48 ± 22.24	28.23 ± 7.58
TN (µg.L^{-1})	347.21 ± 435.45	239.03 ± 124.37	212.24 ± 213.36	254.96 ± 78.32	281.73 ± 81.32	93.22 ± 51.10
